# Associations Between Weight Loss Prior to Knee and Hip Arthroplasty and Peri‐ and Post‐Operative Outcomes: A Systematic Review and Meta‐Analysis

**DOI:** 10.1111/obr.70160

**Published:** 2026-05-12

**Authors:** Moneet Gill, Tim Lindsay, Daniel Kevin Llanera, Tricia M.‐M. Tan, Alex Liddle, Chioma Izzi‐Engbeaya

**Affiliations:** ^1^ MSK Lab, Department of Surgery and Cancer Imperial College London London UK; ^2^ Department of Endocrinology and Diabetes Imperial College Healthcare NHS Trust London UK; ^3^ Section of Investigative Medicine and Endocrinology, Department of Metabolism, Digestion and Metabolism Imperial College London London UK; ^4^ Department of Orthopaedic Surgery Imperial College Healthcare NHS Trust London UK

**Keywords:** arthroplasty, bariatric surgery, joint replacement complications, obesity, weight loss

## Abstract

**Background:**

In some of the existing literature, obesity is associated with increased complications in those who undergo lower limb arthroplasty. We conducted a systematic review and meta‐analysis to assess if pre‐arthroplasty weight loss mitigates increased post‐operative risks.

**Methods:**

This systematic review and meta‐analysis was registered on PROSPERO (CRD42023431110). Key terms were utilized to search PubMed, EMBASE, Ovid, Web of Science, Google Scholar, and Europe PMC. Peer‐reviewed articles published between January 1, 2010, and December 31, 2024, were screened. Studies that reported specific outcomes (superficial wound infection, deep wound infection, pulmonary embolus [PE], deep vein thrombosis [DVT], and revision surgery) in an intervention group (patients with a BMI > 30 kg/m^2^ who underwent pre‐arthroplasty weight loss) and a control group (patients who underwent arthroplasty without preceding weight loss) 90 days post‐arthroplasty were selected and data were extracted from studies meeting these inclusion criteria. Data were analyzed using a random effects model.

**Findings:**

Twenty‐one articles were included in the meta‐analysis. In people with obesity, pre‐arthroplasty weight loss was associated with an increased risk of revision surgery in the first 90 days following lower limb arthroplasty (OR 1.37 [1.01–1.84], *p* = 0.04). Weight loss did not alter the risk of post‐operative DVT, PE, superficial wound infections, and deep wound infections.

**Conclusions:**

Our results demonstrate that pre‐arthroplasty weight loss does not improve short‐term post‐operative outcomes in people with obesity. Large, well‐designed prospective studies should be performed to provide robust data for the development of evidence‐based guidelines for lower limb arthroplasty in people living with obesity.

**Abbreviations**
BMI: body mass index
CI: confidence interval
DVT: deep vein thrombosis
GLP1‐RAs: glucagon‐like peptide 1 receptor agonists
NJR: National Joint Registry
OR: odds ratio
PE: pulmonary embolism
SGLT2is: sodium‐glucose cotransporter 2 inhibitors
VTE: venous thromboembolism
WHO: World Health Organization

## 1 Introduction

Obesity is a significant public health challenge and its prevalence has doubled in adults worldwide since 1990 [1]. With the growing disease burden of obesity, there has been a sharp rise in associated comorbidities [1, 2]. Currently, people living with obesity have a higher incidence of osteoarthritis, cardiovascular disease, diabetes mellitus, obstructive pulmonary disease, cancer, and venous thromboembolism [3, 4], with negative impacts on quality of life and life expectancy.

Osteoarthritis is a complex disease process influenced by both inflammatory and metabolic factors [5]. It is characterized by continuous compression of weight bearing joints, which leads to ongoing articular cartilage and bone damage and remodeling [6]. There is evidence to suggest a pathophysiological relationship between obesity and osteoarthritis [7]. The increased levels of circulating inflammatory markers found in obesity increase the immune response in cartilage, predisposing to osteoarthritis [8]. Furthermore, leptin (produced by adipocytes in proportion to fat mass) has been shown to be associated with reduced cartilage in knee joints [9]. Worryingly, people with obesity (i.e., a body mass index [BMI] ≥ 30 kg/m^2^) have up to a seven times greater likelihood of developing knee osteoarthritis compared with those with a BMI < 25 kg/m^2^ [10]. The risk of hip osteoarthritis is also increased in people living with obesity [11].

Arthroplasty (joint replacement) is used to treat hip and knee osteoarthritis when nonsurgical treatments are unsuccessful, and pain becomes debilitating. Registry data reveal that ~60% of people in the United States and United Kingdom who have knee arthroplasty [12, 13] and ~40% of people who have hip arthroplasty in the United Kingdom have obesity [14]. In contrast to previous guidance, recently updated guidelines recommend that referral for arthroplasty [15] and the timing of arthroplasty should not be delayed to achieve weight loss in people with obesity [16]. However, access to arthroplasty is restricted by funding bodies, which aim to encourage weight loss prior to surgery by enforcing BMI cutoffs [17]. Furthermore, existing guidelines have been based on very low‐quality evidence [16] and the literature consists of conflicting reports about the impact of obesity and weight loss on post‐arthroplasty complications [12, 18, 19, 20, 21, 22]. Analysis of data from the National Joint Registry (NJR) has shown little variability in the outcomes of people with obesity who undergo total knee arthroplasty, with no significant increase in morbidity [13]. Evan et al. have also theorized that data may be skewed due to the obesity paradox, where surgeons operate on patients with obesity who are fitter and/or have fewer comorbidities than people with similar BMIs who are deemed unsuitable for arthroplasty [13]. In light of the high prevalence of obesity and the use of BMI cutoffs to limit access to arthroplasty (which may contribute to health inequalities), it is important to determine if weight loss prior to arthroplasty is associated with improved post‐surgical outcomes in people with obesity. We hypothesized that preoperative weight loss is associated with lower risks of complications following lower limb arthroplasty. To test this hypothesis, we conducted a systematic review and meta‐analysis of studies published between January 1, 2010, and December 1, 2024.

## 2 Material and Methods

### 2.1 Search Strategy

A review of published literature was performed using PubMed, Medline, Ovid, Google Scholar, Web of Science, and Europe PMC databases. Gray literature including Clinicaltrials.gov.uk, the WHO International Clinical Trials Registry Platform (ICTRP), and the European Union Clinical Trials Register were also searched to identify any ongoing research studies. This systematic review was registered a priori on PROSPERO (ID: CRD42023431110).

This systematic review was reported in accordance with the PRISMA checklist (Data S1). The key search terms included variations on the terms “weight loss,” “bariatric surgery,” and “arthroplasty.” These were combined with Boolean operators “AND” and “OR,” as shown in Data S2. The search strategy was modified for each database to ensure that all suitable articles were included in the search results. All articles identified during these searches that were published between January 1, 2010, and December 31, 2024, were screened. Only articles published on or after January 1, 2010, were included to ensure that this systematic review and meta‐analysis were representative of the use of modern prostheses and arthroplasty techniques.

### 2.2 Eligibility Criteria

#### 2.2.1 Inclusion and Exclusion Criteria

Careful definition of the study question and rigorous study selection were performed to ensure a robust systematic review and meta‐analysis [23]. Each article included in the search result was screened using strict inclusion and exclusion criteria independently by two authors (M.G. and D.L.). Primary research articles (cohort studies, randomized controlled trials, registry, and database studies) were included if they had a clearly defined weight loss intervention group and a non‐weight loss comparator group and reported specific outcomes (superficial wound infection, deep wound infection, deep vein thrombosis [DVT], pulmonary embolism [PE], or revision surgery) up to 90 days after hip or knee arthroplasty. Articles for which the full text was unavailable, perspectives, narrative reviews, systematic reviews, and book chapters were excluded. In addition, case reports, case series, letters, editorials, and commentaries were excluded. Articles were reviewed using an online citation manager, Covidence. This allowed automated identification and removal of duplicates and enabled the authors (M.G. and D.L.) to assess each article independently. In the event of a disagreement between M.G. and D.L. about inclusion of an article, a third author (T.L.) independently assessed the article and made the final decision about inclusion or exclusion of the article.

### 2.3 Outcomes

The primary outcome measures were divided into five main groups. These included incidences of surgical site infection, deep prosthetic joint infection, revision surgery, deep vein thrombosis, and pulmonary embolism.

### 2.4 Quality Assessment

An assessment of the bias and suitability of each article was conducted independently by two reviewers (M.G. and D.L.). A third reviewer (T.L.) independently assessed articles for which there was a disagreement. The Downs and Blacks assessment tool was used to rank each of the articles identified [24].

### 2.5 Data Collection and Analysis

Data were extracted by two authors (M.G. and D.L.). The data collated from each study included study design, level of evidence, number of participants, age range, gender, and outcomes measured. A data collection tool was developed using Microsoft Excel, and the meta‐analysis was conducted using Review Manager 2020 [25]. An independent *χ* ^2^ test was conducted to allow comparison of the two groups, “pre‐arthroplasty weight loss” and “control.”

The null hypothesis is that there is no significant reduction in complications when comparing the intervention group that lost weight preoperatively (via either lifestyle, medical, and/or surgical interventions) to the control group who did not lose weight prior to arthroplasty. The null hypothesis is rejected if the *p* value < 0.05. For dichotomous values in this study, odds ratios (OR) with a 95% confidence interval (CI) were calculated using a random effects model. A random effects model was used because it is an appropriate model when comparing studies that are likely to have related but different intervention effects that are assumed to follow a normal distribution [26]. All statistical tests included in this meta‐analysis were two‐tailed. Heterogeneity between studies was assessed using the *I* ^2^ statistic, which highlights the proportion of variance in each data set.

## 3 Results

### 3.1 Study Selection and Characteristics

Five thousand one hundred eighty‐nine articles were identified across all databases: 1502 were removed as duplicates, and 3687 abstracts were reviewed and screened. Eighty‐eight abstracts were then assessed for eligibility using full article screening. Sixty‐seven were excluded at this stage (Data S3). Exclusion reasons included ineligible outcomes, ineligible interventions, ineligible patient populations, full‐text unavailability, or because the articles were not primary research. Therefore, 21 articles were included in the meta‐analysis, and data were extracted from these articles (Figure 1).

**FIGURE 1:**
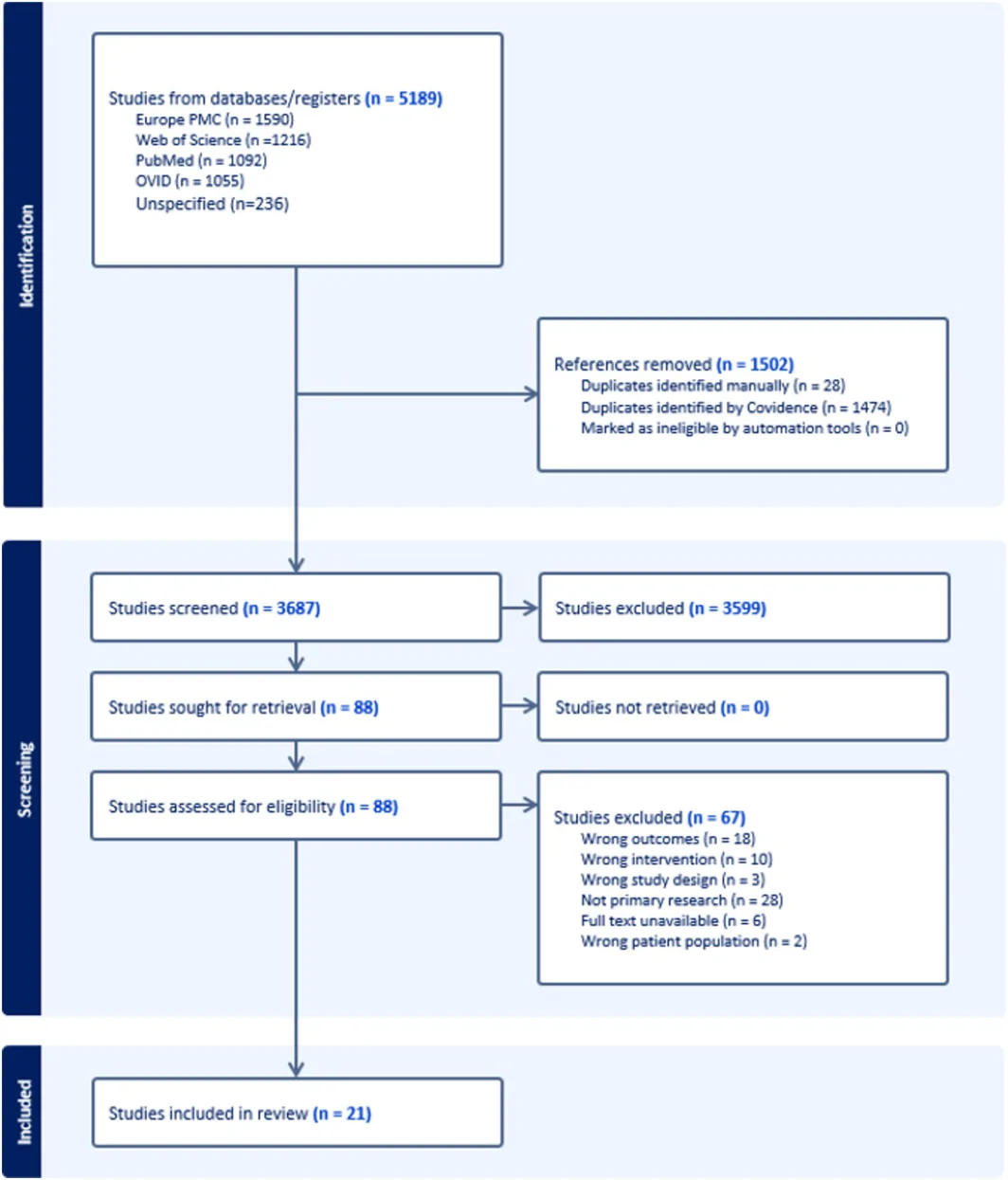
PRISMA flow diagram showing the identification and selection of articles included in the systematic review.

Data extracted from each of the 21 studies included patient groups, outcomes, key results, and limitations. A summary of the characteristics of the included studies, highlighting the types of studies, the year, and country of publication, is outlined in Table 1.

**TABLE 1:** Summary of characteristics of the studies included in the systematic review and meta‐analysis.

Article title	Authors	Year	Study type (country of study population)	Weight loss intervention (I) and control (C) groups
Bariatric Surgery Prior to Total Hip Arthroplasty: Does Timing or Type Matter?	Bains et al. [27]	2022	Retrospective cohort study (USA)	I: Sleeve gastrectomy or Roux‐en‐Y gastric bypass prior to hip arthroplasty C: No history of bariatric surgery before hip arthroplasty
Effect of Bariatric Surgery on Risk of Complications After Total Knee Arthroplasty A Randomized Clinical Trial	Dowsey et al. [28]	2022	Parallel group, assessor‐blinded, randomized clinical trial (Australia)	I: Laparoscopic adjustable gastric banding prior to knee arthroplasty C: Usual care (without bariatric surgery) before knee arthroplasty
A Pilot Study Investigating Dietetic Weight Loss Interventions and 12‐Month Functional Outcomes of Patients Undergoing Total Joint Replacement	Gandler et al. [29]	2016	Randomized controlled trial (Netherlands)	I: Personalized dietary and behavior change advice with ≥ 4 individual support sessions with study dietitian over 1 year prior to lower limb joint arthroplasty C: Usual care
Pain, Function, and Satisfaction After Total Knee Arthroplasty, With or Without Bariatric Surgery	Ighani Arani et al. [30]	2022	Retrospective cohort study (Sweden)	I: Sleeve gastrectomy or gastric bypass prior to knee arthroplasty C: No history of bariatric surgery before knee arthroplasty
Bariatric Surgery Prior to Total Joint Arthroplasty May Not Provide Dramatic Improvements in Post‐Arthroplasty Surgical Outcomes	Inacio et al. [31]	2014	Retrospective single health system cohort study (USA)	I: Bariatric surgery (gastric bypass, gastroplasty, gastric banding, gastric balloon insertion) prior to hip or knee arthroplasty C: Eligible for bariatric surgery but did not have bariatric surgery prior to hip or knee arthroplasty
Preoperative Weight Loss for Morbidly Obese Patients Undergoing Total Knee Arthroplasty: Determining the Necessary Amount	Keeney et al. [18]	2019	Retrospective cohort observational study (USA)	I: Documented weight change within 90 days of knee arthroplasty (weight loss modality not specified) C: Documented weight increase or no weight loss within 90 days of knee arthroplasty
Does Bariatric Surgery Prior to Lower Limb Joint Replacement Reduce Complications?	Kulkarni et al. [32]	2011	Retrospective cohort study (England)	I: Bariatric surgery (gastric stapling, gastric banding, gastric balloon insertion, sleeve gastrectomy or gastric bypass) prior to hip or knee arthroplasty C: Hip or knee arthroplasty prior to bariatric surgery
Preoperative Bariatric Surgery Utilization Is Associated With Increased 90‐Day Postoperative Complication Rates After Total Joint Arthroplasty	Liu et al. [33]	2020	Retrospective cohort analysis (USA)	I: Bariatric surgery (gastric stapling, gastric banding, gastric balloon insertion, sleeve gastrectomy or gastric bypass) prior to hip or knee arthroplasty C: People with or without obesity who did not have bariatric surgery prior to hip or knee arthroplasty
Bariatric Surgery Does Not Improve Outcomes in Patients Undergoing Primary Total Knee Arthroplasty	Martin et al. [34]	2015	Retrospective matched cohort Study (USA)	I: Bariatric surgery (type not specified) prior to knee arthroplasty C: Patients who did not have bariatric surgery with BMI matched to BMI of bariatric surgery group prior to bariatric surgery or matched to BMI of bariatric surgery group after bariatric surgery
Bariatric Surgery Improves Outcomes After Lower Extremity Arthroplasty in the Morbidly Obese: A Propensity Score‐Matched Analysis of a New York Statewide Database	McLawhorn et al. [35]	2018	Propensity matched retrospective cohort study (USA)	I: Bariatric surgery (sleeve gastrectomy, gastric bypass, gastric banding or gastroplasty) prior to hip or knee arthroplasty C: Patients who did not have bariatric surgery with BMI matched to BMI of bariatric surgery group prior to bariatric surgery
Benefits of Bariatric Surgery Before Elective Total Joint Arthroplasty: Is There a Role for Weight Loss Optimization?	Nearing et al. [36]	2017	Retrospective case/cohort study (USA)	I: Bariatric surgery (sleeve gastrectomy or Roux‐en‐Y gastric bypass) prior to hip or knee arthroplasty C: Hip or knee arthroplasty prior to bariatric surgery
Lingering Risk: Bariatric Surgery Before Total Knee Arthroplasty	Nickel et al. [37]	2016	Retrospective cohort (claim based) study (USA)	I: Bariatric surgery (type not specified) prior to knee arthroplasty C: BMI > 40 kg/m^2^ or < 25 kg/m^2^ prior to knee arthroplasty without bariatric surgery
Dislocation Rate Increases With Bariatric Surgery Before Total Hip Arthroplasty	Nickel et al. [38]	2018	Retrospective cohort (claim based) study (USA)	I: Bariatric surgery (type not specified) prior to hip arthroplasty C: BMI > 40 kg/m^2^ or < 25 kg/m^2^ prior to hip arthroplasty without bariatric surgery
Comparison of a Patient‐Centred Weight Loss Program Starting Before Versus After Knee Replacement: A Pilot Study	Pellegrini et al. [39]	2018	Randomized pilot study (USA)	I: 14‐week calorie deficit diet and exercise program started1–6 weeks prior to knee arthroplasty C: 14‐week calorie deficit diet and exercise program started 12 weeks post‐knee arthroplasty
Does Bariatric Surgery Prior to Primary Total Knee Arthroplasty Improve Outcomes?	Ryan et al. [40]	2021	Retrospective cohort study (USA)	I: Bariatric surgery (gastric banding, Roux‐en‐Y gastric bypass, sleeve gastrectomy or unknown procedure type) prior to knee arthroplasty C: BMI ≥ 40 kg/m^2^ or < 40 kg/m^2^ prior to knee arthroplasty without bariatric surgery
Frank Stinchfield Award: Does Bariatric Surgery Prior to Primary Total Hip Arthroplasty Really Improve Outcomes?	Ryan et al. [41]	2022	Retrospective matched cohort study (USA)	I: Bariatric surgery (gastric banding, Roux‐en‐Y gastric bypass, sleeve gastrectomy or unknown procedure type) prior to hip arthroplasty C: BMI ≥ 40 kg/m^2^ or < 40 kg/m^2^ prior to hip arthroplasty without bariatric surgery
Preoperative Weight Loss Before Total Hip Arthroplasty Negatively Impacts Postoperative Outcomes	Schmerler et al. [42]	2024	Retrospective cohort study (USA)	I: Loss of 10% body weight (modality not specified) 6 months before total hip arthroplasty C: No history of weight loss prior to total hip arthroplasty
Total Knee Arthroplasty in Morbidly Obese Patients Treated With Bariatric Surgery: A Comparative Study	Severson et al. [43]	2012	Retrospective case control study (USA)	I: Bariatric surgery (gastric banding or gastric bypass) prior to knee arthroplasty C: Knee arthroplasty prior to bariatric surgery
The Impact of Preoperative Weight Loss Timing on Surgical Outcomes in Total Hip Arthroplasty	Shul et al. [44]	2024	Retrospective cohort study (USA)	I: Documented weight loss from BMI of 40 to 50 kg/m^2^ to < 35 kg/m^2^ in 12 months prior to total hip arthroplasty. C: People with a BMI of 20 to 30 kg/m^2^ and > 40 kg/m^2^ who underwent total hip arthroplasty.
Impact of Bariatric Surgery on Inpatient Complication, Cost, and Length of Stay Following Total Hip or Knee Arthroplasty	Wang et al. [45]	2019	Retrospective (propensity‐matched) cohort study (USA)	I: Bariatric surgery (type not specified) prior to hip or knee arthroplasty in people with BMI ≥ 40 kg/m^2^ C: Knee arthroplasty without prior to bariatric surgery in people with BMI ≥ 40 kg/m^2^
Bariatric Surgery Prior to Total Knee Arthroplasty Is Associated With Fewer Postoperative Complications	Werner et al. [46]	2015	Retrospective cohort study (USA)	I: Patients with BMI > 40 kg/m2 who underwent bariatric surgery (including gastric banding or gastric bypass) prior to knee arthroplasty C: Patients with BMI > 40 kg/m^2^ or < 30 kg/m^2^ prior to knee arthroplasty without bariatric surgery

### 3.2 Quality Assessment

The quality of the included studies was assessed using the Downs and Black criteria [24] as shown in Data S4. Each included study detailed clear hypotheses and aims. The main outcome measures were reliable and valid. Only three of the studies were randomized prospective studies, and only one of these studies included blinding. All of the other included studies were retrospective analyses; therefore, they did not include blinding. The interventions in the studies were either bariatric surgery or a structured weight loss program prior to hip or knee arthroplasty. All 21 studies used appropriate statistical tests. Ten studies did not present raw data that could be used in the meta‐analysis.

### 3.3 Meta‐Analysis

The intervention group consisted of people with an initial BMI ≥ 30 kg/m^2^ undergoing a pre‐arthroplasty weight loss intervention and the comparator (control) group consisted of individuals who did not undergo a weight loss intervention prior to arthroplasty. Data extracted were used to assess the incidence of superficial wound infection, deep wound infection, pulmonary embolism, deep vein thrombosis, and revision surgery occurring within 90 days of knee or hip arthroplasty. The results of the meta‐analysis are summarized in Data S5.

#### 3.3.1 Superficial and Deep Wound Infections

The incidence of superficial wound infection was calculated using 11 studies. In the intervention group, 157 patients developed this complication out of 9742 patients in total. In the control group, 6337 patients developed this complication out of 545,212 patients in total. The rate of deep wound infections was compared across five studies. Of the 6016 patients in the intervention group, 182 developed this complication. There were 465,399 patients in the control group, and 4692 of these had deep wound infections that affected the prosthesis. Pre‐arthroplasty weight loss was not associated with increased or decreased odds of superficial or deep wound infections (Figure 2).

**FIGURE 2:**
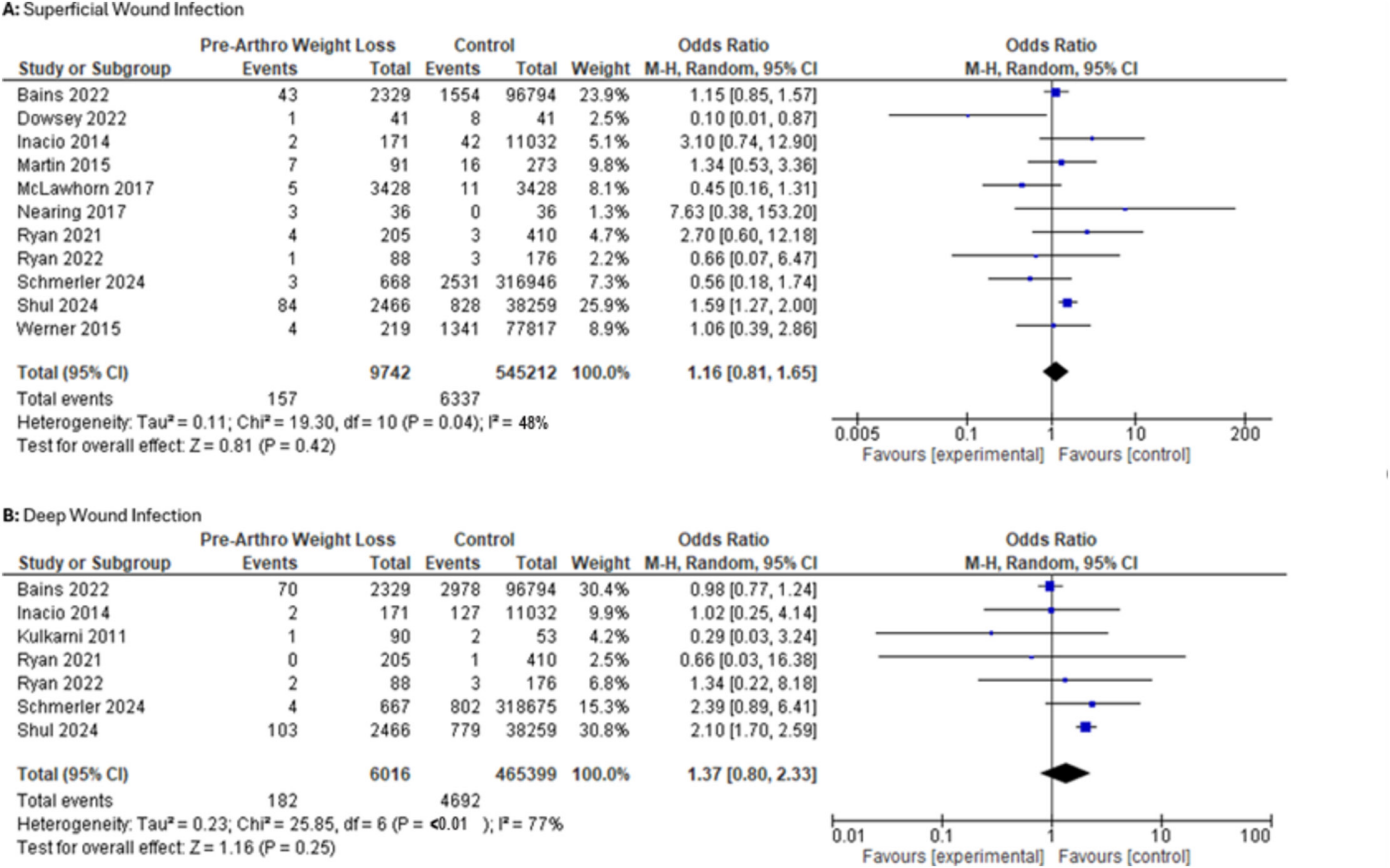
Forest plots showing the effect of pre‐arthroplasty weight loss versus control on the incidence of superficial wound infection (A) or deep wound infection (B). The sizes of the blue squares denote the weight of the study included in the analysis. CI, confidence interval.

#### 3.3.2 Venous Thromboembolism

The incidence of DVT was assessed using data from 13 studies with a total of 16,117 patients in the intervention group and 816,625 people in the control group. There were 439 incidences of DVT in the intervention group and 7871 in the control group. The risk of developing a DVT was not significantly different when comparing the intervention group with the control group (OR 1.13 [95% CI 0.85–1.50], *p* = 0.41). There was high variability between studies as indicated by the *I* ^2^ measure of statistical heterogeneity used here (*I* ^2^ = 57%).

One hundred sixty of the 15,921 patients in the pre‐arthroplasty weight loss group developed a PE. In the control group, 5051 of 816,457 patients developed a PE. Pre‐arthroplasty weight loss was not associated with a lower risk of developing a PE (Figure 3). There was a high degree of statistical heterogeneity between studies (*I* ^2^ = 75%).

**FIGURE 3:**
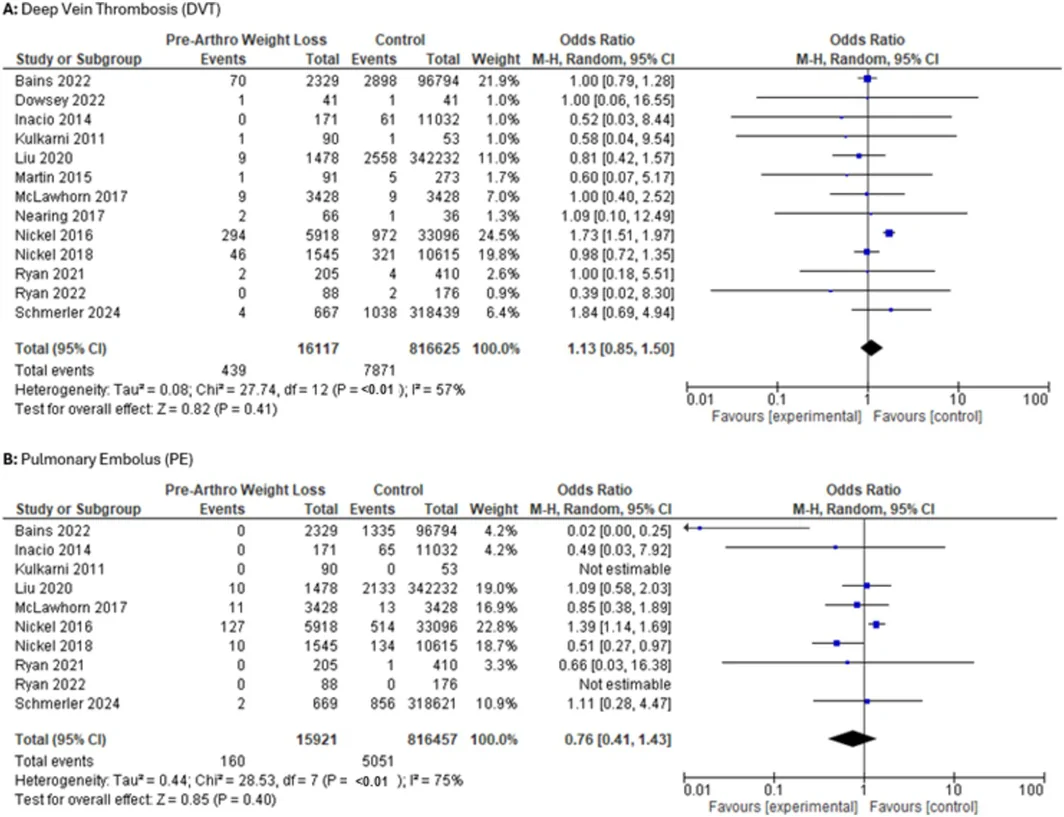
Forest plots showing the effect of pre‐arthroplasty weight loss versus control on the incidence of deep vein thrombosis (A) or pulmonary embolism (B). The sizes of the blue squares denote the weight of the study included in the analysis. CI, confidence interval.

#### 3.3.3 Revision Surgery

Revision surgery data were collated from 11 studies, as shown in Figure 4. There were 12,945 patients in the intervention group and 190,520 patients in the control group. There was an increased risk of revision surgery in the pre‐arthroplasty weight loss group (OR 1.37 [95% CI 1.01–1.84], *p* = 0.04). There was a high degree of statistical heterogeneity between the included studies (*I* ^2^ = 62%).

**FIGURE 4:**
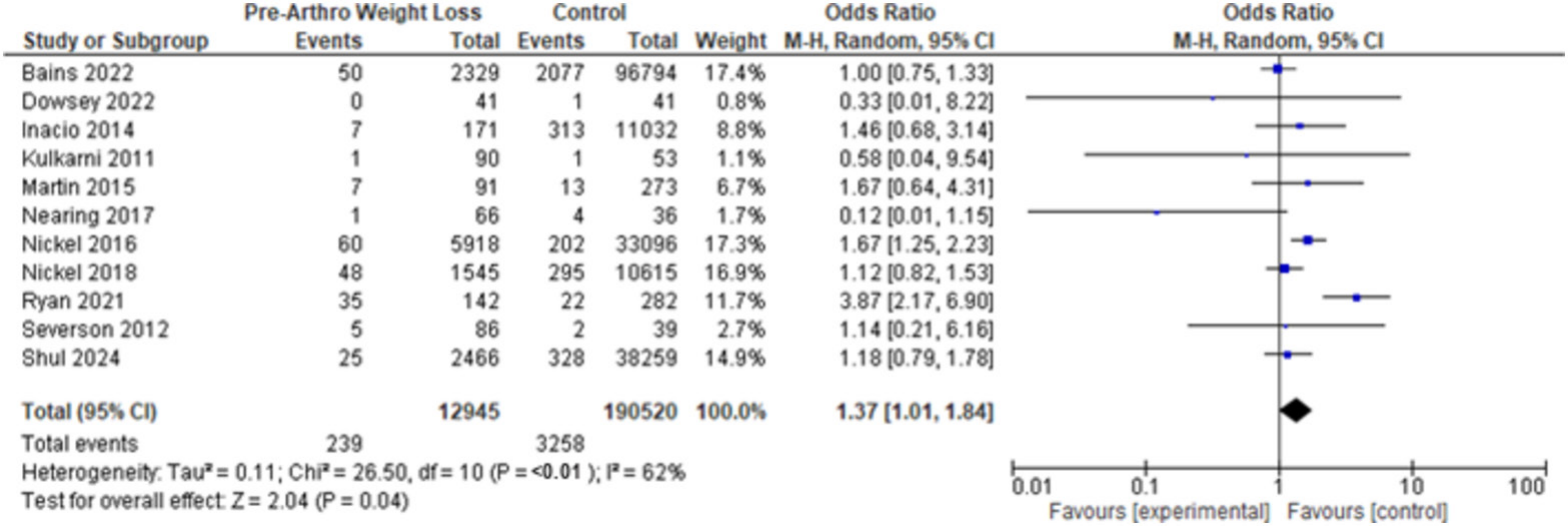
Forest plots showing the effect of pre‐arthroplasty weight loss versus control on the incidence of revision surgery. CI, confidence interval.

## 4 Discussion

Our results demonstrate that there is an increased risk of revision surgery within 90 days of arthroplasty in the intervention group when compared with patients in the control group. In addition, the intervention, preoperative weight loss, did not alter the risks of post‐arthroplasty wound infections or venous thromboembolism. Some recently updated guidelines do not recommend the use of BMI cutoffs for arthroplasty and/or do not recommend delaying arthroplasty to achieve weight loss [15, 16]. They highlight that low‐quality evidence was used to inform these recommendations [15, 16]. However, other guidelines still recommend using BMI for risk stratification [47]. This may significantly impact patients as arthroplasty may be deferred until a BMI that is deemed to confer a lower risk is achieved. Our results indicate that although weight loss may mitigate some risks, pre‐arthroplasty weight loss does not seem to reduce the odds of developing important post‐arthroplasty complications.

A BMI ≥ 30 kg/m^2^ is an independent risk factor for the development of VTE after arthroplasty [48]. In the intervention group, those who lost weight pre‐operatively may still have high BMIs and as such still have greater risks than those in the control group. We must also consider that significant weight loss, either surgical or diet‐based, can be associated with rebound weight gain post‐arthroplasty [49], again increasing the risk of VTE [48]. In our study, the results for the incidence of DVT and PE following surgery were not statistically significant. With improvements in treatments, there is now a focus on early rehabilitation and anticoagulation for proactive prevention of both DVT and PE, and prompt treatment and diagnosis of DVT to prevent PE.

Revision surgery rates were higher in the first 90 days post‐arthroplasty in those who underwent a weight loss intervention. These findings are consistent with those of Kim et al. who have shown that weight loss prior to arthroplasty is an independent variable that leads to an increase in all‐cause revision rates post‐total knee arthroplasty [49]. This could be secondary to poor nutrition and thus poor wound healing due to the preceding weight loss intervention. In addition, revision surgery itself remains a confounding factor, increasing the risk of VTE due to the additional independent risk of thromboembolism when undergoing further surgery [50].

Our results demonstrate that the risk of superficial or deep wound infection in the first 90 days following arthroplasty is similar in the intervention and control groups. This contrasts with findings reported by Kapadia et al. who concluded that obesity was a major cause of increased prosthetic joint infections [51]. Increased body weight was associated with other factors such as increased risk of diabetes, which together increased the overall risk of developing early wound infection either superficial or deep to the joint [51, 52]. Our findings highlight the need for further research in this field.

Patient‐reported outcomes (PROMs) and activity scores following arthroplasty are important post‐arthroplasty outcomes that were not included in our systematic review and meta‐analysis. Ighani Arani et al. investigated the 1‐year patient‐reported pain and activity outcomes in total knee arthroplasty patients [30]. They showed no improvement in recovery time when comparing those who underwent weight loss interventions with a control group, which consisted of BMI‐matched patients [30]. Their findings were echoed by Gandler et al. who concluded that there were no significant differences in pain, function, and activity levels in the two groups following arthroplasty [29]. Pellegrini et al. also showed that there was no improvement in patient outcomes when comparing those who had pre‐arthroplasty weight loss to those who did not [39]. Similar to our findings, these studies do not indicate that there are significant improvements in functionality and outcomes in those who undergo weight loss prior to arthroplasty [19, 53, 54]. This raises important questions about the role of weight loss prior to this life‐altering intervention. Of note, Dowsey et al. have shown that a proportion of those who underwent bariatric surgery did not then go on to have knee arthroplasty due to an improvement in symptoms [28]. It is difficult to assess why this may have been the case, but it raises the question that perhaps those who are fitter and undergo bariatric surgery will ultimately have fewer long‐term complications from associated disease processes such as osteoarthritis.

Our results incorporated data from 21 different studies, resulting in large patient cohorts for both our intervention and control groups. To our knowledge, this is the largest meta‐analysis conducted on this topic and as such provides further insights into this field as a whole. However, there remains a lack of high‐quality data to inform the ongoing debate about the benefits and risks of pre‐arthroplasty weight loss. In our review, we have only included papers published after 2010. This has made the data more representative of modern practices utilizing more advanced surgical techniques in both arthroplasty and bariatric surgery as well as the use of modern joint implants. In addition, there has been ongoing modernization in prevention of wound infections and VTE. Therefore, unlike previous meta‐analyses, our conclusions are relatable to the current health landscape.

One of the key limitations of our study was the lack of differentiation within our intervention group. The data extracted were not sufficiently granular for subgroup analyses to assess the complication rates between different classes of obesity, such as those with a BMI ≥ 40 kg/m^2^ versus those with a BMI between 30 and 39 kg/m^2^. Current literature suggests that there are increased complication rates with a BMI ≥ 40 kg/m^2^ [49] but similar or lower rates of some complications in people with a BMI between 30 and 39 kg/m^2^ compared with people with a BMI < 25 kg/m^2^ [13]. The studies that we included only used bariatric surgery or weight management programs as weight loss interventions, as no studies utilizing weight loss medication met the inclusion criteria. Therefore, we cannot quantify the impact of weight loss medication on our patient population. Glucagon‐like peptide 1 receptor agonists (GLP‐1RAs) and related medications can produce between 15% and 22% weight loss and are increasingly recommended as neoadjuvant interventions to enable critical surgery to be performed [55]. Therefore, studies assessing the impact of their use on post‐arthroplasty outcomes are required. There were large variations in size between the control and intervention groups included in our study, and event rates of complications varied substantially between control groups in different studies. Furthermore, other factors unrelated to weight loss (such as age) may have impacted outcomes.

In most of the included studies, the degree of weight loss was not quantified, the length of time over which weight loss occurred was not reported and/or the time between weight loss and arthroplasty for those in the intervention group was not reported. The absence of these data meant that further detailed analysis into these factors could not be performed. Without knowing how much weight was lost by patients or their starting BMI immediately prior to arthroplasty, we cannot reliably comment on the risk of complications, as we know that these risks are stratified by body weight [13]. We also note that there was a lack of weight matching for subjects in some of the included studies; thus, we were comparing patients who had obesity in the intervention group to patients who had a range of BMIs (from normal to obese) in the control group. This is likely to have contributed to heterogeneity between studies and may limit the generalisability of our results. The high levels of heterogeneity could have been interrogated with the use of detailed sub‐group analysis [56]. However, this was not possible with the included studies.

In addition, our meta‐analysis includes several studies that have been conducted by the same research groups using similar patient data, and most of the included studies were conducted in the United States and involved bariatric surgery‐induced weight loss. This further highlights the need for ongoing research in this field, especially from different geographical regions and utilizing novel weight reducing medication.

Weight loss using bariatric surgery or structured weight loss programs can lead to changes in 90‐day complication rates. However, there are significant gaps in the literature due to the need for adequately powered prospective studies. Future work should focus on quantifying the impact of weight loss secondary to weight management medication and the impact these may have on those undergoing arthroplasty. The use of GLP‐1RAs and sodium‐glucose cotransporter 2 inhibitors (SGLT2is) leads to loss of fat mass; however, they are also associated with the loss of lean body mass and skeletal muscle [57]. The use of weight loss medication may help to reduce BMI prior to arthroplasty, but if there is loss of skeletal muscle mass, this can potentially worsen post‐operative outcomes by delaying recovery and reducing patient satisfaction scores. There has been increasing research into how weight loss can be achieved while preserving muscle mass. For instance, Nunn et al. have shown that blocking the action of activin‐II receptors with bimagrumab can preserve skeletal muscle mass in animal models without compromising the weight loss effect of GLP‐1RAs [58]. The relationship between muscle mass and BMI has also been shown to change as individuals age, especially in post‐menopausal women [59]. This may be a significant confounding factor when assessing post‐arthroplasty complications.

There should also be consideration to the cost‐effectiveness of weight loss prior to arthroplasty, and whether the potential reduction in complication rates can justify the weight loss intervention. Keeney et al. have investigated the impact of weight on total cost of hospital admission for arthroplasty, showing that the biggest driver in increased costs was length of stay. Those who had higher starting weights and BMI ≥ 40 kg/m^2^ had increased length of stay and longer operation times [18]. By contrast, Wang et al. conducted a cost‐analysis which concluded that there was no difference in total hospital cost between the group that underwent arthroplasty post‐bariatric surgery compared with the group that did not have bariatric surgery [45]. However, weight loss in people with obesity has well‐established beneficial effects on multiple (non‐arthroplasty) health indices [60]. Therefore, these conflicting findings highlight the need for more research to conclusively determine the role of weight loss in the optimization of people with obesity prior to arthroplasty, and this should preferably take the form of randomized controlled trials.

## 5 Conclusions

In this meta‐analysis of studies published between 2010 and 2024, weight loss prior to knee or hip arthroplasty was associated with an increased risk of revision surgery in people with obesity, whereas the risks of venous thromboembolism and wound infection were not impacted by weight loss. Uncertainties remain about the impact of pre‐arthroplasty weight loss in people with different obesity classes and the impact of pre‐arthroplasty weight loss on post‐surgical outcomes that were not assessed in this study. Thus, there is a need for studies with BMI‐, age‐, and gender‐matched control groups compared with weight loss intervention groups. Well‐designed prospective studies conducted in different geographical areas are required to provide robust evidence to inform guidelines for lower limb arthroplasty in people with obesity.

## Author Contributions

**Moneet Gill:** data curation, methodology, investigation, formal analysis, data curation, visualization, writing the original draft, and writing review and editing. **Tim Lindsay:** conceptualization, methodology, investigation, formal analysis, supervision, validation, and writing review and editing. **Daniel Kevin Llanera:** investigation and writing review and editing. **Tricia M.‐M. Tan:** methodology, funding acquisition, and writing review and editing. **Alex Liddle:** conceptualization, methodology, supervision, and writing review and editing. **Chioma Izzi‐Engbeaya:** conceptualization, methodology, validation, supervision, and writing review and editing. All authors read and approved the final manuscript. Chioma Izzi‐Engbeaya is the guarantor of this work.

## Funding

The Section of Endocrinology and Investigative Medicine is funded by grants from the MRC and NIHR and is supported by the NIHR Biomedical Research Centre Funding Scheme and the NIHR/Imperial Clinical Research Facility. The following authors are also funded: T.L. is funded by an NIHR Academic Clinical Fellowship and Imperial College London. T.M.‐M.T. is funded by the NIHR Imperial Biomedical Research Centre, NIHR Efficacy and Mechanism Evaluation Scheme, Diabetes UK, Novo Nordisk UK Research Foundation, and Leadiant Pharmaceutials. C.I. is funded by an NIHR Senior Clinical and Practitioner Research Award and an NIHR Imperial BRC Pilot Grant.

## Conflicts of Interest

CI has received consultancy fees from Novo Nordisk and Boehringer Ingelheim unrelated to this work. All other authors have no conflicts of interest to declare.

## Supporting information


**Data S1:** PRISMA Checklist.
**Data S2: Key Search Terms:** Search Strategy used for the systematic review.
**Data S3: Excluded Studies:** References for excluded papers as defined in the PRISMA flowsheet.
**Data S4: Assessment of the Quality of Included Studies:** Risk of bias in included studies. Abbreviations: Y, Yes; N, No; UTD Unable to Determine.
**Data S5: Results of the Meta‐analysis:** Summary of results produced from a random effect model meta‐analysis. Data are presented as Odds Ratio (with 95% CI) alongside a *p* value and the raw figures for each intervention. Abbreviations: CI, Confidence Interval.

## Data Availability

Data are available online (https://zenodo.org/records/14905630).
