# Supplementary material for: Associations Between Weight Loss Prior to Knee and Hip Arthroplasty and Peri‐ and Post‐Operative Outcomes: A Systematic Review and Meta‐Analysis
Source: Obes Rev. 2026 May 12;27(12):e70160. doi: 10.1111/obr.70160 (PMC13749250; doi:10.1111/obr.70160)
Supplement: Supplementary file 1 [file OBR-27-e70160-s001.pdf]

Associations between weight loss prior to knee and hip arthroplasty and peri- and post-operative outcomes: A systematic review and meta-analysis

Running Title: Pre-arthroplasty weight loss and post-operative outcomes

Moneet Gill[a], Tim Lindsay[a], Daniel Kevin Llanera[b], Tricia M-M Tan[b,c], Alex Liddle[a,d], Chioma Izzi-Engbeaya[b,c]

[a]MSK lab, Department of Surgery and Cancer, Imperial College London, London, UK, W12 0BZ

[b]Department of Endocrinology and Diabetes, Imperial College Healthcare NHS Trust, London, UK, W2 1NY

[c]Section of Investigative Medicine and Endocrinology, Department of Metabolism, Digestion and Metabolism, Imperial College London, London, UK, W12 0NN

[d]Department of Orthopaedic Surgery, Imperial College Healthcare NHS Trust, London, UK, W2 1NY

Supplementary Appendix A: PRISMA Checklist

Section and Topic	Item #	Checklist item	Location where item is reported
**TITLE**			
Title	1	Identify the report as a systematic review.	1
**ABSTRACT**			
Abstract	2	See the PRISMA 2020 for Abstracts checklist.	2-3
**INTRODUCTION**			
Rationale	3	Describe the rationale for the review in the context of existing knowledge.	5-6
Objectives	4	Provide an explicit statement of the objective(s) or question(s) the review addresses.	6
**METHODS**			
Eligibility criteria	5	Specify the inclusion and exclusion criteria for the review and how studies were grouped for the syntheses.	7
Information sources	6	Specify all databases, registers, websites, organisations, reference lists and other sources searched or consulted to identify studies. Specify the date when each source was last searched or consulted.	6
Search strategy	7	Present the full search strategies for all databases, registers and websites, including any filters and limits used.	6 and Appendix B
Selection process	8	Specify the methods used to decide whether a study met the inclusion criteria of the review, including how many reviewers screened each record and each report retrieved, whether they worked independently, and if applicable, details of automation tools used in the process.	8

Section and Topic	Item #	Checklist item	Location where item is reported
Data collection process	9	Specify the methods used to collect data from reports, including how many reviewers collected data from each report, whether they worked independently, any processes for obtaining or confirming data from study investigators, and if applicable, details of automation tools used in the process.	8
Data items	10a	List and define all outcomes for which data were sought. Specify whether all results that were compatible with each outcome domain in each study were sought (e.g. for all measures, time points, analyses), and if not, the methods used to decide which results to collect.	8
	10b	List and define all other variables for which data were sought (e.g. participant and intervention characteristics, funding sources). Describe any assumptions made about any missing or unclear information.	8
Study risk of bias assessment	11	Specify the methods used to assess risk of bias in the included studies, including details of the tool(s) used, how many reviewers assessed each study and whether they worked independently, and if applicable, details of automation tools used in the process.	8
Effect measures	12	Specify for each outcome the effect measure(s) (e.g. risk ratio, mean difference) used in the synthesis or presentation of results.	9
Synthesis methods	13a	Describe the processes used to decide which studies were eligible for each synthesis (e.g. tabulating the study intervention characteristics and comparing against the planned groups for each synthesis (item #5)).	9 + Table 1
	13b	Describe any methods required to prepare the data for presentation or synthesis, such as handling of missing summary statistics, or data conversions.	9
	13c	Describe any methods used to tabulate or visually display results of individual studies and syntheses.	9
	13d	Describe any methods used to synthesize results and provide a rationale for the choice(s). If meta-analysis was performed, describe the	9

Section and Topic	Item #	Checklist item	Location where item is reported
		model(s), method(s) to identify the presence and extent of statistical heterogeneity, and software package(s) used.	
	13e	Describe any methods used to explore possible causes of heterogeneity among study results (e.g. subgroup analysis, meta-regression).	9
	13f	Describe any sensitivity analyses conducted to assess robustness of the synthesized results.	9
Reporting bias assessment	14	Describe any methods used to assess risk of bias due to missing results in a synthesis (arising from reporting biases).	8
Certainty assessment	15	Describe any methods used to assess certainty (or confidence) in the body of evidence for an outcome.	8
**RESULTS**			
Study selection	16a	Describe the results of the search and selection process, from the number of records identified in the search to the number of studies included in the review, ideally using a flow diagram.	9 + Figure 1
	16b	Cite studies that might appear to meet the inclusion criteria, but which were excluded, and explain why they were excluded.	Appendix B
Study characteristics	17	Cite each included study and present its characteristics.	Table 1
Risk of bias in studies	18	Present assessments of risk of bias for each included study.	Appendix D
Results of	19	For all outcomes, present, for each study: (a) summary statistics for each group (where appropriate) and (b) an effect estimate and its precision	10-11 +

Section and Topic	Item #	Checklist item	Location where item is reported
individual studies		(e.g. confidence/credible interval), ideally using structured tables or plots.	Figures 2-4
Results of syntheses	20a	For each synthesis, briefly summarise the characteristics and risk of bias among contributing studies.	Appendix D
	20b	Present results of all statistical syntheses conducted. If meta-analysis was done, present for each the summary estimate and its precision (e.g. confidence/credible interval) and measures of statistical heterogeneity. If comparing groups, describe the direction of the effect.	10-11 + Appendix E
	20c	Present results of all investigations of possible causes of heterogeneity among study results.	10-11
	20d	Present results of all sensitivity analyses conducted to assess the robustness of the synthesized results.	10-11
Reporting biases	21	Present assessments of risk of bias due to missing results (arising from reporting biases) for each synthesis assessed.	Appendix D
Certainty of evidence	22	Present assessments of certainty (or confidence) in the body of evidence for each outcome assessed.	10-11 + Appendix D
**DISCUSSION**			
Discussion	23a	Provide a general interpretation of the results in the context of other evidence.	12-16
	23b	Discuss any limitations of the evidence included in the review.	14-15
	23c	Discuss any limitations of the review processes used.	14-15
	23d	Discuss implications of the results for practice, policy, and future research.	14-17
**OTHER INFORMATION**			

Section and Topic	Item #	Checklist item	Location where item is reported
Registration and protocol	24a	Provide registration information for the review, including register name and registration number, or state that the review was not registered.	6
	24b	Indicate where the review protocol can be accessed, or state that a protocol was not prepared.	6
	24c	Describe and explain any amendments to information provided at registration or in the protocol.	n/a
Support	25	Describe sources of financial or non-financial support for the review, and the role of the funders or sponsors in the review.	17
Competing interests	26	Declare any competing interests of review authors.	17
Availability of data, code and other materials	27	Report which of the following are publicly available and where they can be found: template data collection forms; data extracted from included studies; data used for all analyses; analytic code; any other materials used in the review.	18 + Table 1

Supplementary Appendix B: Key Search Terms

Weight Loss	**AND**	(arthroplasty **OR** hip replacement **OR** knee replacement)
(bariatric surgery **OR** sleeve gastrectomy **OR** Roux-en-Y gastric bypass **OR** gastric bypass **OR** gastric band **OR** gastroplasty **OR** gastric balloon)	**AND**	(arthroplasty **OR** hip replacement **OR** knee replacement)

Supplementary Appendix C: Excluded Studies

	Reference	Reason for Exclusion
**1**	Ao Y, Wang F, Yang L. A commentary on "Does bariatric surgery really benefit patients before total knee arthroplasty? A systematic review and meta-analysis" (Int. J. Surg. 2022;104:106778). International Journal of Surgery. 2022;106:106927. DOI: https://doi.org/10.1016/j.ijsu.2022.106927	Not Primary Research
**2**	Benotti PN, Still CD, Craig Wood G, Seiler JL, Seiler CJ, Thomas SP, et al. Surgical weight-loss to improve functional status trajectories following total knee arthroplasty: SWIFT trial: Rationale, design, and methods. Contemp Clin Trials. 2018;69:1-9. DOI: 10.1016/j.cct.2018.03.012	Not Primary Research
**3**	Bookman JS, Schwarzkopf R, Rathod P, Iorio R, Deshmukh AJ. Obesity: The Modifiable Risk Factor in Total Joint Arthroplasty. Orthopedic Clinics of North America. 2018;49(3):291-6. DOI: https://doi.org/10.1016/j.ocl.2018.02.002	Not Primary Research
**4**	Burkard T, Holmberg D, Wretenberg P, Thorell A, Hügle T, Burden AM. The associations between bariatric surgery and hip or knee arthroplasty, and hip or knee osteoarthritis: Propensity score-matched cohort studies. Osteoarthr Cartil Open. 2022;4(2):100249. DOI: 10.1016/j.ocarto.2022.100249	Wrong Outcomes
**5**	Chen X. Comment on 'Does bariatric surgery really benefit patients before total knee arthroplasty? a systematic review and meta-analysis'. International Journal of Surgery. 2023;109(5).	Not Primary Research
**6**	Coelho, A., Leal-Blanquet, J., Sánchez-Soler, J.F. *et al*. Patients lose weight after a total knee arthroplasty: myth or reality?. *International Orthopaedics (SICOT)* 46, 1299–1304 (2022). https://doi-org.iclibezp1.cc.ic.ac.uk/10.1007/s00264-022-05387-0	Wrong Outcomes
**7**	Crookes PF, Cassidy RS, Machowicz A, et al. Should isolated morbid obesity influence the decision to operate in hip and knee arthroplasty?. *Bone Jt Open*. 2021;2(7):515-521. doi:10.1302/2633-1462.27.BJO-2021-0062.R1	Wrong Intervention
**8**	Dowsey MM, Liew D, Stoney JD, Choong PF. The impact of pre-operative obesity on weight change and outcome in total knee replacement. *J Bone Joint Surg Br*. 2010;92-B(4):513-520. doi:10.1302/0301-620X.92B4.23174	Wrong Intervention
**9**	Feng JE, Novikov D, Anoushiravani AA, Schwarzkopf R. Total knee arthroplasty: improving outcomes with a multidisciplinary approach. *J Multidiscip Healthc*. 2018;11:63-73 https://doi.org/10.2147/JMDH.S140550	Not Primary Research
**10**	Fournier MN, Hallock J, Mihalko WM. Preoperative Optimization of Total Joint Arthroplasty Surgical Risk: Obesity. J Arthroplasty. 2016;31(8):1620-4. DOI: 10.1016/j.arth.2016.02.085	Not Primary Research
**11**	Gu A, Cohen JS, Malahias M-A, Lee D, Sculco PK, McLawhorn AS. The Effect of Bariatric Surgery Prior to Lower-Extremity Total Joint Arthroplasty: A Systematic Review. HSS Journal®. 2019;15(2):190-200. doi:10.1007/s11420-019-09674-2	Not Primary Research
**12**	Jester R, Rodney A. The relationship between obesity and primary Total Knee Replacement: A scoping review of the literature. International Journal of Orthopaedic and Trauma Nursing. 2021;42:100850. DOI: https://doi.org/10.1016/j.ijotn.2021.100850	Not Primary Research
**13**	Kostic AM, Leifer VP, Selzer F, Hunter DJ, Paltiel AD, Chen AF, et al. Cost-Effectiveness of Weight-Loss Interventions Prior to Total Knee Replacement for Patients With Class III Obesity. Arthritis Care Res (Hoboken). 2023;75(8):1752-63. DOI: 10.1002/acr.25044	Wrong Outcomes
**14**	Kunutsor, S.K., Whitehouse, M.R. & Blom, A.W. Obesity paradox in joint replacement for osteoarthritis — truth or paradox?. *GeroScience* 44, 651–659 (2022). https://doi.org/10.1007/s11357-021-00442-x	Not Primary Research
**15**	Li S, Luo X, Sun H, Wang K, Zhang K, Sun X. Does Prior Bariatric Surgery Improve Outcomes Following Total Joint Arthroplasty in the Morbidly Obese? A Meta-Analysis. J Arthroplasty. 2019 Mar;34(3):577-585. doi: 10.1016/j.arth.2018.11.018.	Not Primary Research
**16**	Lyons, M. Melanie PhD, MSN-ACNP-BC; Walsh, Colleen MS; Sheth, Neil P. MD; Williams, Noel N. MD; Gurubhagavatula, Indira MD, MPH; Mihalko, William M. MD. Obesity, Orthopaedics, and Outcomes. Journal of the American Academy of Orthopaedic Surgeons 23(4):p 210-212, April 2015. | DOI: 10.5435/JAAOS-D-15-00098	Not Primary Research
**17**	Martin, J. Ryan MD; Jennings, Jason M. MD, DPT; Dennis, Douglas A. MD. Morbid Obesity and Total Knee Arthroplasty: A Growing Problem. Journal of the American Academy of Orthopaedic Surgeons 25(3):p 188-194, March 2017. | DOI: 10.5435/JAAOS-D-15-00684	Not Primary Research
**18**	Matziolis G, Brodt S. Total Hip Arthroplasty in Young and Obese Patients. Z Orthop Unfall. 2020;158(02):184-7. DOI: 10.1055/a-0989-2765	Full Text Unavailable
**19**	McLaughlin J, Kipping R, Owen-Smith A, McLeod H, Hawley S, Wilkinson JM, Judge A. What effect have NHS commissioners' policies for body mass index had on access to knee replacement surgery in England?: An interrupted time series analysis from the National Joint Registry. PLoS One. 2022 Jun 29;17(6):e0270274. doi: 10.1371/journal.pone.0270274. PMID: 35767546; PMCID: PMC9242471.	Wrong Outcomes
**20**	Mellion KM, Grover BT. Obesity, Bariatric Surgery, and Hip/Knee Arthroplasty Outcomes. Surgical Clinics of North America. 2021;101(2):295-305. DOI: https://doi.org/10.1016/j.suc.2020.12.011	Not Primary Research
**21**	Müller M, Gutwerk A, Greve F, Völker L, Zyskowski M, Kirchhoff C, Biberthaler P, Pförringer D, Braun K. The Association between High Body Mass Index and Early Clinical Outcomes in Patients with Proximal Femur Fractures. *Journal of Clinical Medicine*. 2020; 9(7):2076. https://doi.org/10.3390/jcm9072076	Wrong Patient Population
**22**	Peiris CL, Leahy E, Galletti J, Taylor NF. Multidisciplinary Intervention Before Joint Replacement Surgery May Improve Outcomes for People with Osteoarthritis and Metabolic Syndrome: A Cohort Study. Metabolic Syndrome and Related Disorders. 2021;19(8):428-35. DOI: 10.1089/met.2021.0029	Full Text Unavailable

	Reference	Reason for Exclusion		Reference	Reason for Exclusion
23	Rahman, Tahsin M. BS; Fleifel, Dominik BA; Padela, Muhammad T. MD; Anoushiravani, Afshin MD; Rizvi, Syed Ahmad; El-Othmani, Mouhanad M. MD; Sayeed, Zain MD, MHA. Interventions for Obesity and Nutritional Status in Arthroplasty Patients. JBJS Reviews 8(3):p e0161, March 2020. | DOI: 10.2106/JBJS.RVW.19.00161	Not Primary Research	32	Wu M, Cochrane NH, Kim B, Belay ES, O'Donnell J, Ryan SP, et al. Patterns and Predictors of Weight Change Before and After Total Hip Arthroplasty in Class 2 and 3 Obese Patients. The Journal of Arthroplasty. 2022;37(5):880-7. DOI: 10.1016/j.arth.2022.01.009	Wrong Outcomes
24	Schwartz, Adam J. MD1, a. CORRInsights®: Bariatric Orthopaedics: Total Knee Arthroplasty in Super-obese Patients (BMI > 50 kg/m2). Survivorship and Complications. Clinical Orthopaedics and Related Research 471(11):p 3531-3532, November 2013. | DOI: 10.1007/s11999-013-3221-2	Not Primary Research	33	Yayac M, Aggarwal R, Parvizi J. How viable is pre-surgery weight reduction for the reduction of periprosthetic joint infection risk after total joint arthroplasty? Expert Review of Medical Devices. 2020;17(3):149-51. DOI: 10.1080/17434440.2020.1720509	Not Primary Research
25	Schwarzkopf R, Lavery JA, Hooper J, Parikh M, Gold HT. Bariatric Surgery and Time to Total Joint Arthroplasty: Does It Affect Readmission and Complication Rates? Obes Surg. 2018 May;28(5):1395-1401. DOI: 10.1007/s11695-017-3034-6. PMID: 29168111.	Wrong Outcomes	34	Long-Term Clinical and Economic Implications of Surgical Weight Loss Interventions for Obese Patients With Knee Osteoarthritis. Lippincott's Bone and Joint Newsletter. 2023;29(2).	Full Text Unavailable
26	Seward MW, Briggs LG, Bain PA, Chen AF. Preoperative Nonsurgical Weight Loss Interventions Before Total Hip and Knee Arthroplasty: A Systematic Review. The Journal of Arthroplasty. 2021;36(11):3796-806.e8. DOI: 10.1016/j.arth.2021.06.021	Not Primary Research	35	2023 BESOBAJS. Knee. Bone & Joint. 2023;2023(15/8/2023). DOI: 10.1302/2048-0105.122.360046	Full Text Unavailable
27	Seward MW, Chen AF. Obesity, preoperative weight loss, and telemedicine before total joint arthroplasty: a review. Arthroplasty. 2022;4(1):2. DOI: 10.1186/s42836-021-00102-7	Not Primary Research	36	Drolson AW. Medically Supervised Weight Loss and Total Knee Arthroplasty. J Arthroplasty. 2024 Dec;39(12):e72. doi: 10.1016/j.arth.2024.04.086. PMID: 39523051.	Wrong Study Design
28	Springer BD, Carter JT, McLawhorn AS, Scharf K, Roslin M, Kallies KJ, et al. Obesity and the role of bariatric surgery in the surgical management of osteoarthritis of the hip and knee: a review of the literature. Surgery for Obesity and Related Diseases. 2017;13(1):111-8. DOI: 10.1016/j.soard.2016.09.011	Not Primary Research	37	McLaughlin, J., Kipping, R., McLeod, H. et al. Health optimisation for patients with obesity before elective orthopaedic surgery: a qualitative study of professionals' views on restrictive approaches and future practice. Perioper Med 13, 104 (2024). https://doi.org/10.1186/s13741-024-00460-1	Wrong Outcome
29	Stets K, Koehler SM, Bronson W, Chen M, Yang K, Bronson M. Weight and body mass index change after total joint arthroplasty. Orthopedics. 2010;33(6):386.	Full Text Unavailable	38	Tidd JL, Huffman N, Oyem PC, Pasqualini I, Hadad MJ, Klika AK, Deren ME, Piuzzi NS. Preoperative and Postoperative Weight Change has Minimal Influence on Health Care Utilization and Patient-Reported Outcomes Following Total Knee Arthroplasty. J Knee Surg. 2024 Jun;37(7):545-554. doi: 10.1055/a-2232-7657. Epub 2023 Dec 19. PMID: 38113913.	Wrong Study Design
30	Wan Y. A commentary on "Does bariatric surgery really benefit patients before total knee arthroplasty? A systematic review and meta-analysis" (Int J Surg 2022;104:106778). International Journal of Surgery. 2022;106:106928. DOI: https://doi.org/10.1016/j.ijsu.2022.106928	Not Primary Research	39	De Mauro D, Balato G, Festa E, Di Cristo A, Marasco L, Loffredo G, Di Lauro P, Di Gennaro D, Maccauro G, Rosa D. Role of bariatric surgery in reducing periprosthetic joint infections in total knee arthroplasty. A systematic review and meta-analysis. BMC Musculoskelet Disord. 2024 Apr 1;25(1):248. doi: 10.1186/s12891-024-07288-2. PMID: 38561717; PMCID: PMC10983737.	Not Primary Research
31	Watts CD, Wagner ER, Houdek MT, Osmon DR, Hanssen AD, Lewallen DG, Mabry TM. Morbid obesity: a significant risk factor for failure of two-stage revision total knee arthroplasty for infection. J Bone Joint Surg Am. 2014 Sep 17;96(18):e154. DOI: 10.2106/JBJS.M.01289. PMID: 25232084.	Wrong Outcomes	40	Hoffmeister, Ellen. Bariatric Surgery and Its Longer-Term Effect on Osteoarthritis and Hip and Knee Arthroplasty. Lippincott's Bone and Joint Newsletter 29(6):p 61-63, June 2023. | DOI: 10.1097/01.BONEJ.0000937148.44903.42	Not Primary Research

	Reference	Reason for Exclusion		Reference	Reason for Exclusion
**41**	Ishimoto R, Mutsuzaki H, Shimizu Y, Yoshikawa K, Koseki K, Takeuchi R, Matsumoto S, Hada Y. Association between Obesity and Short-Term Patient-Reported Outcomes following Total Knee Arthroplasty: A Retrospective Cohort Study in Japan. J Clin Med. 2024 Feb 24;13(5):1291. doi: 10.3390/jcm13051291. PMID: 38592115; PMCID: PMC10932041.	Wrong Outcome	**50**	Sequeira SB, Scuderi GR, Mont MA. Obesity and Concomitant Weight Change Remain Challenging Comorbidities for Arthroplasty Surgeons to Manage. J Arthroplasty. 2024 Jun;39(6):1397-1398. doi: 10.1016/j.arth.2024.04.011. Epub 2024 Apr 5. PMID: 38583681.	Not Primary Research
**42**	Kim BI, Khilnani TK, LaValva SM, Goodman SM, Della Valle AG, Lee GC. Utilization of Glucagon-Like Peptide-1 Receptor Agonist at the Time of Total Hip Arthroplasty for Patients Who Have Morbid Obesity. J Arthroplasty. 2024 Dec 9:S0883-5403(24)01288-9. doi: 10.1016/j.arth.2024.12.008. Epub ahead of print. PMID: 39662850.	Wrong Intervention	**51**	Lohmander LS, Peltonen M, Andersson-Assarsson JC, Maglio C, Sjöholm K, Taube M, Jacobson P, Svensson PA, Carlsson LMS, Ahlin S. Bariatric surgery, osteoarthritis and arthroplasty of the hip and knee in Swedish Obese Subjects - up to 31 years follow-up of a controlled intervention study. Osteoarthritis Cartilage. 2023 May;31(5):636-646. doi: 10.1016/j.joca.2022.11.015. Epub 2023 Feb 6. PMID: 36754250.	Wrong Outcomes
**43**	Genel, F., Pavlovic, N., Gao, M., Hackett, D., Lewin, A., Piya, M., Mills, K., Brady, B., Dennis, S., Boland, R., Harris, I. A., Flood, V. M., Adie, S., & Naylor, J. M. (2024). Optimising body weight in people with obesity prior to knee or hip arthroplasty: A feasibility study utilising a dietitian-led low inflammatory diet. *Musculoskeletal Care*, e1867. https://doi.org/10.1002/msc.1867	Wrong Outcome	**52**	Palacios, Pablo & Palacios, Isabel & Palacios, Ana & Lorente, Alejandro & Mariscal, Gonzalo & Benlloch, María & Palacios, José. (2024). Effect of bariatric surgery on postoperative outcomes of total hip arthroplasty: An updated systematic review and meta-analysis. Asian journal of surgery. 10.1016/j.asjsur.2024.11.003.	Not Primary Research
**44**	Heo KY, Goel RK, Woltemath A, Fuqua A, Hrudka BT, Syed O, Arellano E, Premkumar A, Wilson JM. Glucagon-Like Peptide-1 Receptor Agonist Use Is Not Associated With Increased Complications After Total Hip Arthroplasty in Patients Who Have Type 2 Diabetes. J Arthroplasty. 2024 Oct 30:S0883-5403(24)01139-2. doi: 10.1016/j.arth.2024.10.099. Epub ahead of print. PMID: 39486470.	Wrong Outcome	**53**	A266 Outcomes for knee arthroplasty candidates who undergo bariatric surgery: interim analysis of SWIFT trial results (This research was conducted with support by the Investigator-Initiated Study Program (Grant #ETH-14-621) of Ethicon Endo-Surgery, Inc.). Surgery for Obesity and Related Diseases. 2024;20(6):S1-S2	Wrong Outcomes
**45**	Lau, L.C.M., Chan, P.K., Lui, T.W.D. *et al.* Preoperative weight loss interventions before total hip and knee arthroplasty: a systematic review of randomized controlled trials. *Arthroplasty* **6**, 30 (2024). https://doi.org/10.1186/s42836-024-00252-4	Not Primary Research	**54**	Pavlovic N, Harris IA, Boland R, Brady B, Genel F, Naylor J. The effect of body mass index and preoperative weight loss in people with obesity on postoperative outcomes to 6 months following total hip or knee arthroplasty: a retrospective study. Arthroplasty. 2023 Oct 1;5(1):48. doi: 10.1186/s42836-023-00203-5. PMID: 37777817; PMCID: PMC10544191.	Wrong Intervention
**46**	Liu, Pan BSa; Meng, Jiahao BSa; Tang, Hang BSa; Xiao, Yifan BSa; Li, Xi BSa; Wu, Yumei BSa; Liu, Weijie BSa; Xiong, Yilin MDa; Gao, Shuguang MDa,b,c,d,*. Association between bariatric surgery and outcomes of total joint arthroplasty: a meta-analysis. International Journal of Surgery 111(1):p 1541-1546, January 2025. | DOI: 10.1097/JS9.0000000000002002	Not Primary Research	**55**	LaValva SM, Grubel J, Ong J, Chiu YF, Lyman S, Mandl LA, Cushner FD, Gonzalez Della Valle A, Parks ML. Substantial Weight Loss May Not Improve Early Outcomes of Total Knee Arthroplasty in the Morbidly Obese. J Arthroplasty. 2024 Sep;39(9):2272-2279.e1. doi: 10.1016/j.arth.2024.04.015. Epub 2024 Apr 25. PMID: 38670174.	Full text not available
**47**	LaValva, S. M., et al. (2024). "Is Preoperative Weight Reduction in Patients Who Have Body Mass Index ≥ 40 Associated With Lower Complication Rates After Primary Total Hip Arthroplasty?" The Journal of Arthroplasty **39**(9): S73-S79.	Wrong Outcomes	**56**	de Ree RHGM, de Jong LD, Hazebroek EJ, Somford MP. Optimal timing of hip and knee arthroplasty after bariatric surgery: A systematic review. J Clin Orthop Trauma. 2024 Apr 26;52:102423. doi: 10.1016/j.jcot.2024.102423. PMID: 38766387; PMCID: PMC11096744.	Not Primary Research
**48**	Magruder ML, Miskiewicz MJ, Rodriguez AN, Mont MA. Semaglutide Use Prior to Total Hip Arthroplasty Results in Fewer Postoperative Prosthetic Joint Infections and Readmissions. J Arthroplasty. 2024 Mar;39(3):716-720. doi: 10.1016/j.arth.2023.12.023. Epub 2023 Dec 19. PMID: 38122837.	Wrong Intervention	**57**	Rullán PJ, Oyem PC, Pumo TJ, Khan ST, Pasqualini I, Klika AK, Barsoum WK, Molloy RM, Piuzzi NS. A Longitudinal analysis of weight changes before and after total hip arthroplasty: Weight trends, patterns, and predictors. Technol Health Care. 2024;32(5):3747-3760. doi: 10.3233/THC-231404. PMID: 39331055.	Wrong Intervention
**49**	Mayfield CK, Mont MA, Lieberman JR, Heckmann ND. Medical Weight Optimization for Arthroplasty Patients: A Primer of Emerging Therapies for the Joint Arthroplasty Surgeon. J Arthroplasty. 2024 Jan;39(1):38-43. doi: 10.1016/j.arth.2023.07.017. Epub 2023 Jul 31. PMID: 37531983.	Wrong Intervention	**58**	Magaldi RJ, Strecker SE, Witmer D. Glucagon-Like Peptide-1 Agonists in Total Hip Arthroplasty: Complications, Readmissions, and Patient-Reported Outcomes. J Am Acad Orthop Surg Glob Res Rev. 2024 Nov 15;8(11):e24.00148. doi: 10.5435/JAAOSGlobal-D-24-00148. PMCID: PMC11573327.	Wrong Intervention

	Reference	Reason for Exclusion		Reference	Reason for Exclusion
**59**	Salis Z, Sainsbury A. Association Between Change in Body Mass Index and Knee and Hip Replacements: A Survival Analysis of Seven to Ten Years Using Multicohort Data. Arthritis Care Res (Hoboken). 2023 Jun;75(6):1340-1350. doi: 10.1002/acr.25021. Epub 2023 Jan 5. PMID: 36106942; PMCID: PMC10953021.	Wrong Intervention	**67**	Momtaz DA, Pereira DE, Singh A, Gonuguntla R, Mittal MM, Torres B, Lee TM, Dayhim F, Hosseinzadeh P, Bendich I. Prior Bariatric Surgery Is Associated With Improved Total Hip Arthroplasty Outcomes in Patients Who Have Obesity: A National Cohort Study With 6 Years of Follow-Up. J Arthroplasty. 2025 Jan;40(1):160-168. doi: 10.1016/j.arth.2024.06.065. Epub 2024 Jul 3. PMID: 38969294.	Wrong Outcome
**60**	Kubsad S, Raftis DA, Agarwal AR, Marrache M, Cohen JS, Thakkar SC, Golladay GJ. No Difference in Revision Rates up to 10 years Following Total Hip Arthroplasty in Patients Who Had Prior Bariatric Surgery When Compared to Patients Who Had Class III Obesity: A Propensity Matched Analysis. J Arthroplasty. 2024 Jun;39(6):1399-1403.e1. doi: 10.1016/j.arth.2024.02.065. Epub 2024 Feb 27. PMID: 38423258.	Wrong Study Design			
**61**	Arani, Perna & Wretenberg, Per & Stenberg, Erik & Ottosson, Johan & W-Dahl, Annette. (2022). Total Knee Arthroplasty and Bariatric Surgery: Change in BMI and Risk of Revision Depending on Sequence of Surgery. 10.21203/rs.3.rs-1976145/v1.	Wrong Outcomes			
**62**	Rechenmacher AJ, Yancy WS Jr, Bolognesi MP, Jiranek WA, Seyler TM, Horn ME. Does Preoperative Weight Loss Within 6 Months or 1 Year Change the Risk of Adverse Outcomes in Total Knee Arthroplasty by Initial Body Mass Index Classification? J Arthroplasty. 2023 Dec;38(12):2517-2522.e2. doi: 10.1016/j.arth.2023.06.023. Epub 2023 Jun 16. PMID: 37331436.	Wrong Intervention			
**63**	Sattari SA, Sattari AR, Salib CG, Salem HS, Hameed D, Dubin J, Mont MA. Total Knee Arthroplasty With or Without Prior Bariatric Surgery: A Systematic Review and Meta-Analysis. J Arthroplasty. 2024 Nov;39(11):2863-2871. doi: 10.1016/j.arth.2024.05.034. Epub 2024 May 17. PMID: 38761894.	Not Primary Research			
**64**	Stambough, Jeffrey B. MD. In Patients with Knee OA and Severe Obesity, Bariatric Surgery and Weight Loss Before TKA Reduced Complications Versus TKA Alone. The Journal of Bone and Joint Surgery 105(10):p 805, May 17, 2023. | DOI: 10.2106/JBJS.23.00114	Wrong Outcome			
**65**	Hameed D, Bains SS, Dubin JA, Shul C, Chen Z, Stein A, Nace J, Mont MA. Timing Matters: Optimizing the Timeframe for Preoperative Weight Loss to Mitigate Postoperative Infection Risks in Total Knee Arthroplasty. J Arthroplasty. 2024 Jun;39(6):1419-1423.e1. doi: 10.1016/j.arth.2023.12.028. Epub 2023 Dec 21. PMID: 38135167.	Wrong Outcome			
**66**	Ailaney N, Guirguis PG, Ginnetti JG, et al. Sleeve gastrectomy prior to primary total hip and total knee arthroplasty is associated with increased risk of early complications. Bone Joint J. 2024;106-B(9):935-941. doi:10.1302/0301-620X.106B9.BJJ-2023-1369.R1	Wrong Patient Population			
**67**	Rechenmacher AJ, Yancy WS Jr, Bolognesi MP, Ryan SP, Jiranek WA, Horn ME. Does Medically Supervised Weight Loss Prior to Total Knee Arthroplasty Improve Patient-Reported Pain and Physical Function? J Arthroplasty. 2024 Feb;39(2):350-354. doi: 10.1016/j.arth.2023.08.038. Epub 2023 Aug 18. PMID: 37597821.	Wrong Patient Population			

Supplementary Appendix D: Assessment of the Quality of Included Studies

	Bains et al 2022 [46]	Dowsey et al 2022 [38]	Gandler et al 2016 [34]	I.Arani et al 2022 [33]	Inacio et al 2014 [47]	Keeney et al 2019 [18]	Kulkarni et al 2011 [48]	Liu et al 2020 [49]	Martin et al 2015 [50]	McLawhorn et al 2018 [51]	Nearing et al 2017 [52]	Nickel et al 2016 [53]	Nickel et al 2018 [54]	Pellegrini et al 2018 [35]	Ryan et al 2021 [55]	Ryan et al 2022 [56]	Schmerler et al. 2024 [57]	Severson et al 2012 [58]	Shul et al 2024 [59]	Wang et al 2019 [60]	Werner et al 2015 [61]
Hypothesis/aims/objectives clearly stated	Y	Y	Y	Y	Y	Y	Y	Y	Y	Y	Y	Y	Y	Y	Y	Y	Y	Y	Y	Y	Y
Main outcome measures clearly described.	Y	Y	Y	Y	Y	Y	Y	Y	Y	Y	Y	Y	Y	Y	Y	Y	Y	Y	Y	Y	Y
Characteristics of patients/subjects clearly described	Y	Y	Y	Y	Y	Y	Y	Y	Y	Y	Y	Y	Y	Y	Y	Y	Y	Y	Y	Y	Y
Interventions of interest clearly described	Y	Y	Y	Y	Y	Y	Y	Y	Y	Y	Y	Y	Y	Y	Y	Y	Y	Y	Y	Y	Y
Distribution of principal confounders in each group clearly described	Y	Y	UTD	Y	Y	Y	Y	Y	Y	Y	Y	Y	Y	UTD	Y	Y	Y	Y	Y	Y	Y
Main findings clearly described	Y	Y	Y	Y	Y	Y	Y	Y	Y	Y	Y	Y	Y	Y	Y	Y	Y	Y	Y	Y	Y
Estimates of random variability in the data provided	Y	Y	Y	Y	Y	Y	Y	Y	Y	Y	Y	Y	Y	Y	Y	Y	Y	Y	Y	Y	Y
Important adverse events reported	Y	Y	Y	Y	Y	Y	Y	Y	Y	Y	Y	Y	UTD	Y	Y	Y	Y	Y	Y	Y	Y
Characteristics of patients lost to follow-up described	UTD	Y	UTD	Y	UTD	UTD	UTD	UTD	UTD	N	N	UTD	UTD	Y	UTD	UTD	UTD	UTD	UTD	UTD	UTD
Actual probability values reported	Y	Y	Y	Y	N	Y	Y	Y	Y	Y	Y	Y	Y	Y	Y	Y	Y	Y	Y	Y	Y
Participants approached representative of entire population	Y	Y	Y	Y	Y	Y	Y	Y	Y	Y	Y	Y	N	N	Y	Y	Y	Y	Y	Y	Y
Participants recruited representative of entire population	Y	Y	Y	Y	Y	Y	Y	Y	Y	Y	Y	Y	N	N	N	Y	Y	Y	Y	Y	Y
Staff, places and facilities representative of majority of population	Y	Y	UTD	Y	Y	Y	Y	Y	Y	UTD	Y	Y	N	N	N	Y	Y	Y	Y	Y	Y
Blinding of study subjects	N	N	N	N	N	N	N	N	N	N	N	N	N	N	N	N	N	N	N	N	N
Blinding of assessors	N	Y	N	N	N	N	N	N	N	N	N	N	N	N	N	N	N	N	N	N	N
Data based on data-dredging clearly stated	N/A	N/A	N/A	N/A	N/A	N/A	N/A	N/A	N/A	N/A	N/A	N/A	N/A	N/A	N/A	N/A	N/A	N/A	N/A	N/A	N/A
Adjustment of different length of follow-up or duration between case and control	UTD	Y	UTD	N	N	Y	Y	UTD	Y	Y	UTD	Y	N	UTD	Y	Y	Y	Y	N	UTD	Y
Appropriate statistical tests used.	Y	Y	Y	Y	Y	Y	Y	Y	Y	Y	Y	Y	Y	Y	Y	Y	Y	Y	Y	Y	Y
Compliance to intervention reliable.	Y	UTD	Y	UTD	Y	Y	Y	Y	Y	UTD	Y	Y	Y	Y	Y	Y	Y	Y	Y	Y	Y
Main outcome measure reliable and valid	Y	Y	Y	Y	Y	Y	Y	Y	Y	Y	Y	Y	Y	Y	Y	Y	Y	Y	Y	Y	Y
Intervention groups or case-controls recruited from same population	Y	Y	Y	Y	Y	Y	Y	Y	N	Y	Y	Y	Y	Y	Y	Y	Y	Y	Y	Y	Y
Intervention groups or case-controls recruited at the same time.	Y	Y	Y	Y	Y	Y	Y	Y	N	Y	Y	Y	Y	Y	Y	Y	Y	Y	Y	Y	Y
Study subjects randomized to the interventions	N	Y	Y	N	N	N	N	N	N	N	N	N	N	Y	N	N	N	N	N	N	N
Was concealed randomization to allocation undertaken	N	N	UTD	N	N	N	N	N	N	N	N	N	N	UTD	N	N	N	N	N	N	N
Adequate adjustment made in the analysis of confounders	Y	Y	Y	Y	Y	Y	UTD	UTD	Y	Y	Y	Y	Y	Y	Y	Y	Y	Y	Y	Y	Y
Patient losses accounted for	UTD	UTD	Y	Y	Y	Y	UTD	Y	UTD	UTD	UTD	Y	UTD	Y	UTD	Y	UTD	UTD	UTD	UTD	UTD
Sufficiently powered cohort size	N	N/Y	N	N	N	N	N	N	N	N	N	N	N	N	N	N	N	N	N	N	N

Supplementary Appendix E: Results of the Meta-analysis

Outcome: 90 days post-arthroplasty	Odds Ratio (95% CI)	P value	Events in intervention	Total in intervention	% events/total intervention	Events in control group	Total in control group	% events/total control	Statistical heterogeneity (I^2; chi^2 p value)
Superficial Wound Infection	1.16 (0.81 to 1.65)	0.42	157	9,742	1.61	6,337	545,212	1.16	48%, 0.04
Deep Wound Infection	1.37 (0.80 to 2.33)	0.25	182	6,016	3.02	4,692	465,399	1.00	77%, <0.01
Deep Vein Thrombosis	1.13 (0.85 to 1.50)	0.41	439	16,117	2.72	7,871	816,625	0.96	57%, <0.01
Pulmonary embolism	0.76 (0.41 to 1.43)	0.40	160	15,921	1.00	5,051	816,457	0.62	75%, <0.01
Revision surgery	1.37 (1.01 to 1.84)	0.04	239	12,945	1.85	3,258	190,520	1.71	62%, <0.01
